# Publisher Correction: A dietary fatty acid counteracts neuronal mechanical sensitization

**DOI:** 10.1038/s41467-020-17869-z

**Published:** 2020-08-04

**Authors:** Luis O. Romero, Rebeca Caires, Alec R. Nickolls, Alexander T. Chesler, Julio F. Cordero-Morales, Valeria Vásquez

**Affiliations:** 1 71S. Manassas St. Department of Physiology, College of Medicine, University of Tennessee Health Science Center, Memphis, TN 38103 USA; 2 Integrated Biomedical Sciences Graduate Program, College of Graduate Health Sciences, Memphis, TN 38103 USA; 3 National Center for Complementary and Integrative Health, National Institutes of Health, Bethesda, MD 20892 USA; 4 National Institute of Neurological Disorders and Stroke, National Institutes of Health, Bethesda, MD 20892 USA

**Keywords:** Fatty acids, Ion transport, Physiology, Neurophysiology

Correction to: *Nature Communications*10.1038/s41467-020-16816-2, published online 19 June 2020.

The original version of this article omitted the following from the Acknowledgements:

… R01GM125629…

This has now been corrected in both the PDF and HTML versions of the article.

