# Supplementary material for: A dietary fatty acid counteracts neuronal mechanical sensitization
Source: Nat Commun. 2020 Jun 19;11:2997. doi: 10.1038/s41467-020-16816-2 (PMC7305179; doi:10.1038/s41467-020-16816-2)
Supplement: Supplementary file 1 — Supplementary Information [file 41467_2020_16816_MOESM1_ESM.pdf]

SUPPLEMENTARY INFORMATION

A DIETARY FATTY ACID COUNTERACTS NEURONAL MECHANICAL SENSITIZATION

Romero et al.

Supplementary Figure 1

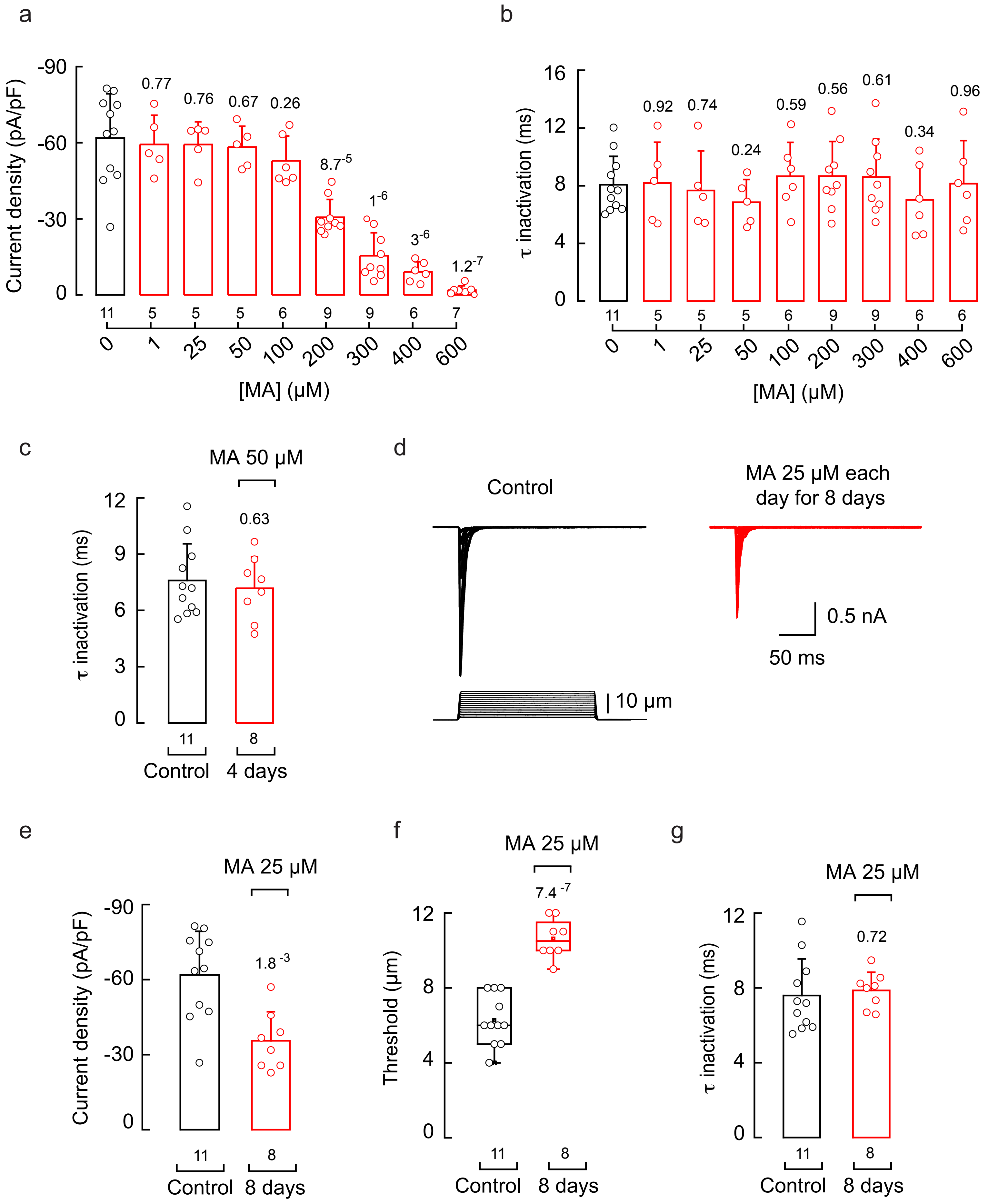

Supplementary figure 1. Related to figure 1.

a Current densities elicited by maximum displacement of control and MA (1, 25, 50, 100, 200, 300, 400, and 600 µM)-treated N2A$^{Piezo1-/-}$ cells transfected with *Piezo2* Variant (V2). Bars are mean ± SD. n is denoted above the *x*-axis. Two tailed unpaired t-test.

b PIEZO2 V2 time constants of inactivation elicited by maximum displacement of control and MA (1, 25, 50, 100, 200, 300, 400, and 600 µM)-treated N2A$^{Piezo1-/-}$ cells. Bars are mean ± SD. n is denoted above the *x*-axis. Two-sided permutation t-test.

c PIEZO2 V2 time constant of inactivation elicited by maximum displacement of control and MA (50 µM each day for 4 days)-treated N2A$^{Piezo1-/-}$ cells. Bars are mean ± SD. n is denoted above the *x*-axis. Two-tailed unpaired t-test.

d Representative currents (at -60 mV) of control and MA (25 µM each day for 8 days)-treated N2A$^{Piezo1-/-}$ cells transfected with *Piezo2* V2.

e PIEZO2 V2 current densities elicited by maximum displacement of control and MA (25 µM each day for 8 days)-treated N2A$^{Piezo1-/-}$ cells. Error bars represent SD. n is denoted above the *x*-axis. Two-tailed unpaired t-test.

f Displacement thresholds required to elicit PIEZO2 V2 currents of control and MA (25 µM each day for 8 days)-treated N2A$^{Piezo1-/-}$ cells transfected with *Piezo2* V2. Boxplots show mean (square), median (bisecting line), bounds of box (75th to 25th percentiles), outlier range with 1.5 coefficient (whiskers), and minimum and maximum data points. n is denoted above the *x*-axis. Two-tailed unpaired t-test.

g PIEZO2 V2 time constant of inactivation elicited by maximum displacement of control and MA (25 µM each day for 8 days)-treated N2A$^{Piezo1-/-}$ cells. Bars are mean ± SD. n is denoted above the *x*-axis. Two-tailed unpaired t-test.

p values are denoted above the bars and box.

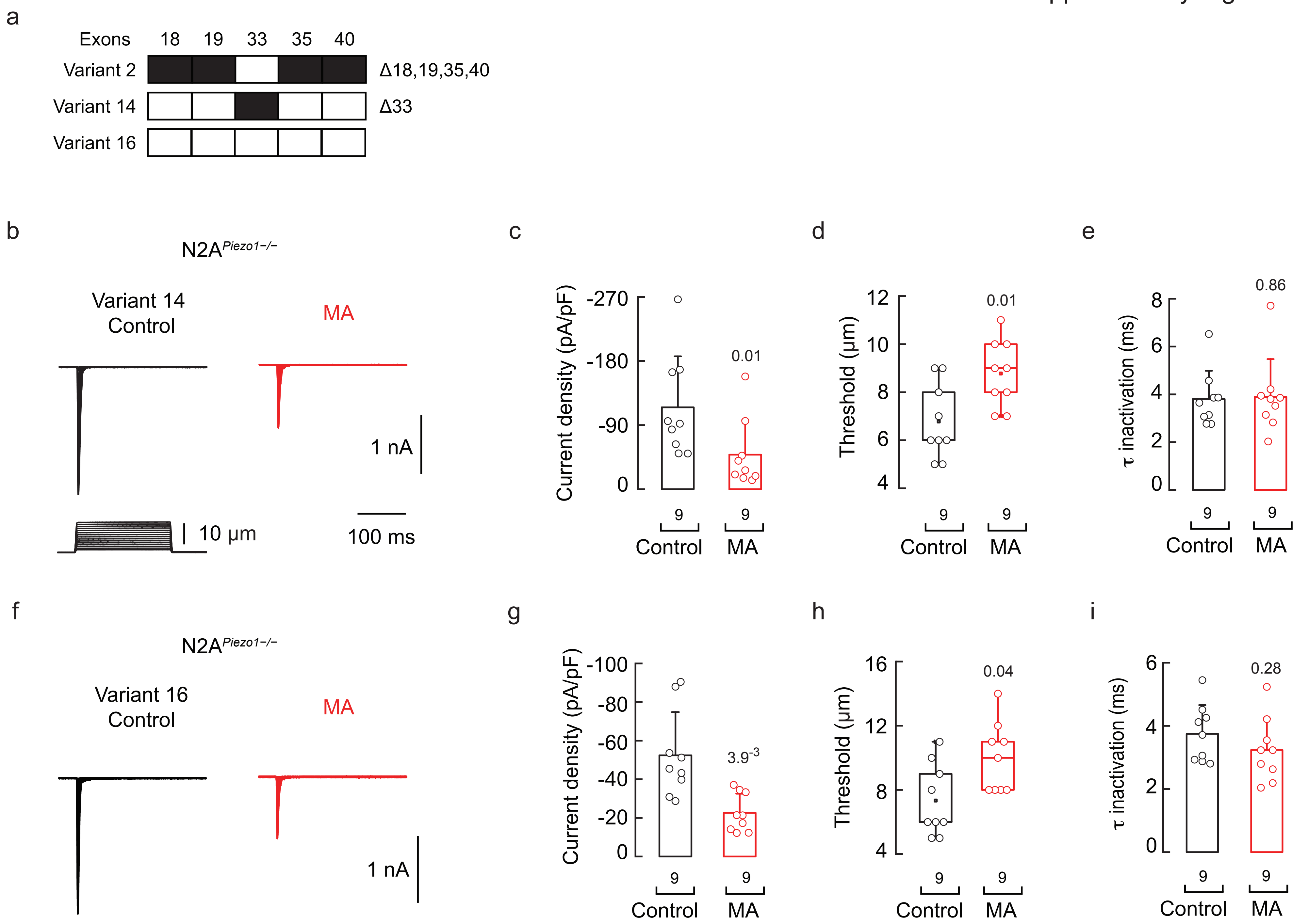
a
Exons 18 19 33 35 40
Variant 2
Δ18,19,35,40
Variant 14
Δ33
Variant 16
b
N2A Piezo1−/−
Variant 14
Control
MA
1 nA
10 μm
100 ms
c
Current density (pA/pF)
-270
-180
-90
0
0.01
9
9
Control
MA
d
Threshold (μm)
12
10
8
6
4
0.01
9
9
Control
MA
e
τ inactivation (ms)
8
6
4
2
0
0.86
9
9
Control
MA
f
N2A Piezo1−/−
Variant 16
Control
MA
1 nA
g
Current density (pA/pF)
-100
-80
-60
-40
-20
0
3.9-3
9
9
Control
MA
h
Threshold (μm)
16
12
8
4
0.04
9
9
Control
MA
i
τ inactivation (ms)
6
4
2
0
0.28
9
9
Control
MA

Supplementary figure 2. Related to figure 1.

a Schematic representation of absent (black) and/or present (white) exons of *Piezo2* variants (V).

b Representative whole-cell patch-clamp recordings of control and MA (300 µM; 18 h)-treated N2A$^{Piezo1-/-}$ cells transfected with *Piezo2* V14.

c Current densities elicited by maximum displacement of control and MA (300 µM; 18 h)-treated N2A$^{Piezo1-/-}$ cells transfected with *Piezo2* V14. Bars are mean ± SD. n is denoted above the *x*-axis. Two-tailed Mann-Whitney test.

d Displacement thresholds required to elicit currents of control and MA (300 µM; 18 h)-treated N2A$^{Piezo1-/-}$ cells transfected with *Piezo2* V14. Boxplots show mean (square), median (bisecting line), bounds of box (75th to 25th percentiles), outlier range with 1.5 coefficient (whiskers), and minimum and maximum data points. n is denoted above the *x*-axis. Two-tailed unpaired t-test.

e Time constants of inactivation elicited by maximum displacement of control and MA (300 µM; 18h)-treated N2A$^{Piezo1-/-}$ cells transfected with *Piezo2* V14. Bars are mean ± SD. n is denoted. Two-tailed Mann-Whitney test.

f Representative whole-cell patch-clamp recordings of control and MA (300 µM; 18 h)-treated N2A$^{Piezo1-/-}$ cells transfected with *Piezo2* V16.

g Current densities elicited by maximum displacement of control and MA (300 µM; 18 h)-treated N2A$^{Piezo1-/-}$ cells transfected with *Piezo2* V16. Bars are mean ± SD. n is denoted above the *x*-axis. Two-tailed unpaired t-test with Welch's correction.

h Displacement thresholds required to elicit currents of control and MA (300 µM; 18 h)-treated N2A$^{Piezo1-/-}$ cells transfected with *Piezo2* V16. Boxplots show mean (square), median (bisecting line), bounds of box (75th to 25th percentiles), outlier range with 1.5 coefficient (whiskers), and minimum and maximum data points. n is denoted above the *x*-axis. Two-tailed Mann-Whitney test.

i Time constants of inactivation elicited by maximum displacement of control and MA (300 µM; 18h)-treated N2A$^{Piezo1-/-}$ cells transfected with *Piezo2* V16. Bars are mean ± SD. n is denoted above the *x*-axis. Two-tailed unpaired t-test.

p values are denoted above the bars and boxes.

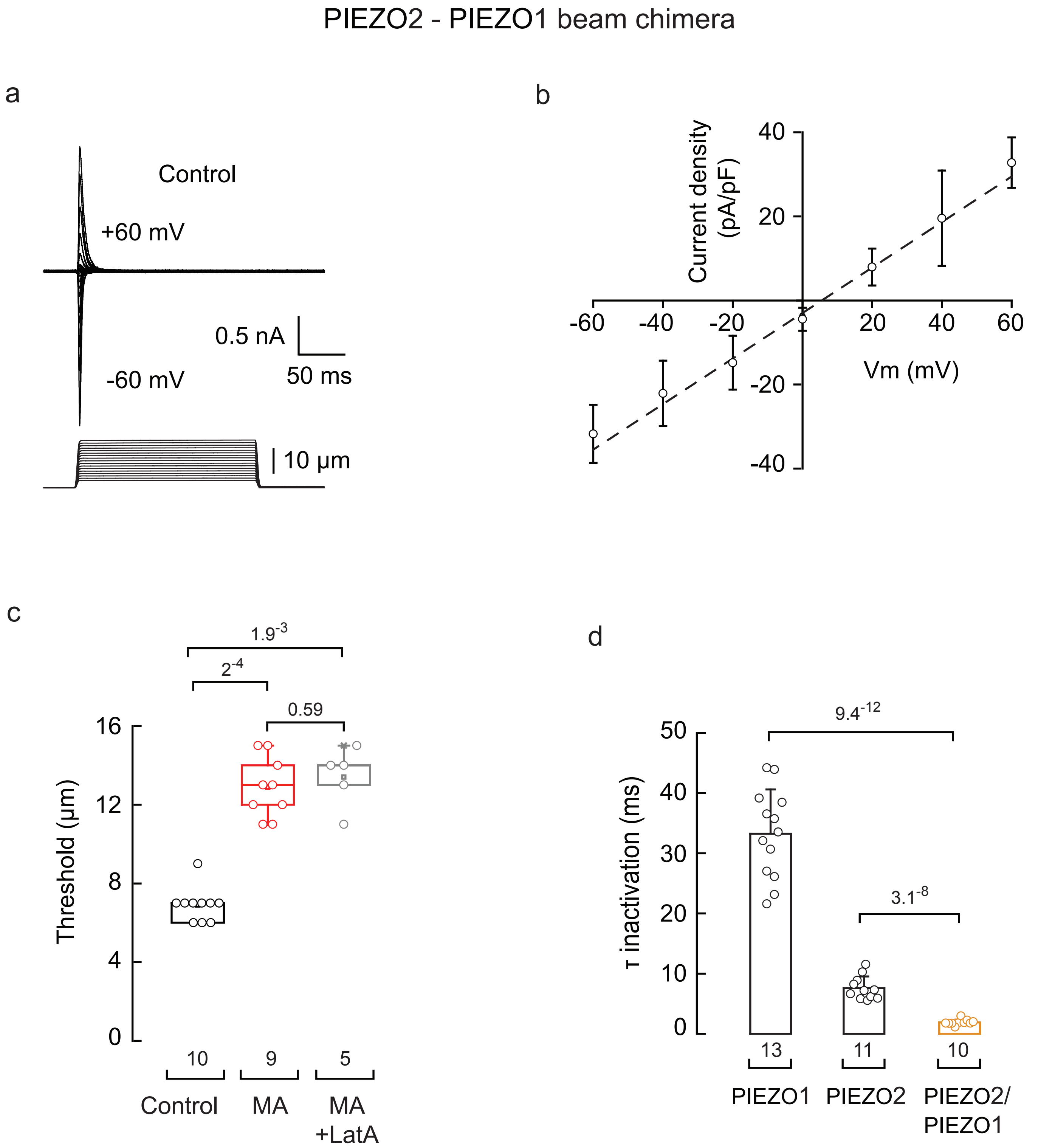
PIEZO2 - PIEZO1 beam chimera
a
Control
+60 mV
0.5 nA
50 ms
-60 mV
10 µm
b
Current density
(pA/pF)
40
20
-20
-40
-60
-40
-20
20
40
60
Vm (mV)
c
1.9-3
2-4
0.59
Threshold (µm)
16
12
8
4
0
10
9
5
Control
MA
MA
+LatA
d
9.4-12
3.1-8
τ inactivation (ms)
50
40
30
20
10
0
13
11
10
PIEZO1
PIEZO2
PIEZO2/
PIEZO1

Supplementary figure 3. Related to figure 2.

a Representative whole-cell patch-clamp recordings elicited by mechanical stimulation (at ± 60 mV) of N2A$^{Piezo1-/-}$ cells transfected with *Piezo2-Piezo1* beam chimera.

b Current-voltage relationship of PIEZO2-PIEZO1 chimera mechano-dependent currents as determined by whole-cell patch-clamp experiments. Circles are mean ± SD. n = 6.

c Displacement thresholds required to elicit currents of control, MA (100 μM for 18 h)-treated N2A$^{Piezo1-/-}$ cells transfected with *Piezo2-Piezo1* beam chimera, with and without Latrunculin A (1 μM for 1 h). Boxplots show mean (square), median (bisecting line), bounds of box (75th to 25th percentiles), outlier range with 1.5 coefficient (whiskers), and minimum and maximum data points. n is denoted above the *x*-axis. Two-tailed Mann-Whitney test (for control *vs.* MA and control *vs*. MA + LatA) and two-tailed unpaired t-test (for MA *vs.* MA + LatA).

d Time constant of inactivation of currents elicited by maximum displacement of N2A cells (expressing endogenous *Piezo1*) and N2A$^{Piezo1-/-}$ cells transfected with *Piezo2* and *Piezo2-Piezo1* beam chimera. Bars are mean ± SD. n is denoted above the *x*-axis. Two-tailed unpaired t-test.

p values are denoted above the boxes and bars.

Supplementary Figure 4

PIEZO1 - PIEZO2 beam chimera

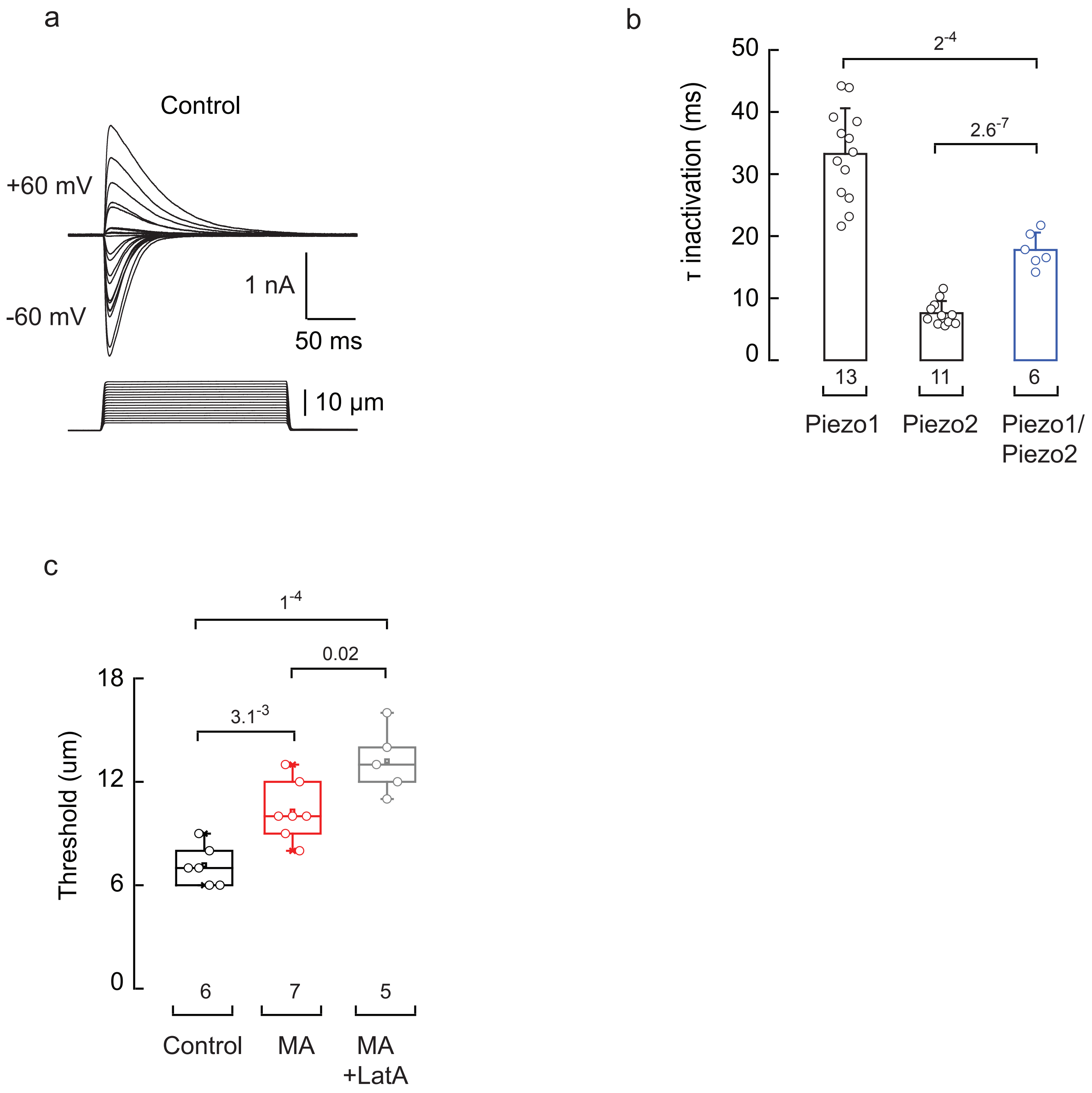

Supplementary figure 4. Related to figure 2.

a Representative whole-cell patch-clamp recordings elicited by mechanical stimulation (at ± 60 mV) of N2A$^{Piezo1-/-}$ cells transfected with *Piezo1-Piezo2* beam chimera.

b Time constant of inactivation of currents elicited by maximum displacement of N2A cells (expressing endogenous *Piezo1*) and N2A$^{Piezo1-/-}$ cells transfected with *Piezo2* and *Piezo1-Piezo2* beam chimera. Bars are mean ± SD. n is denoted above the *x*-axis. Two-tailed unpaired t-test.

c Displacement thresholds required to elicit currents of control, MA (100 µM for 18 h)-treated N2A$^{Piezo1-/-}$ cells transfected with *Piezo1-Piezo2* beam chimera, with and without Latrunculin A (1 µM for 1 h). Boxplots show mean (square), median (bisecting line), bounds of box (75th to 25th percentiles), outlier range with 1.5 coefficient (whiskers), and minimum and maximum data points. n is denoted above the *x*-axis. Two-tailed unpaired t-test.

p values are denoted above the bars and boxes.

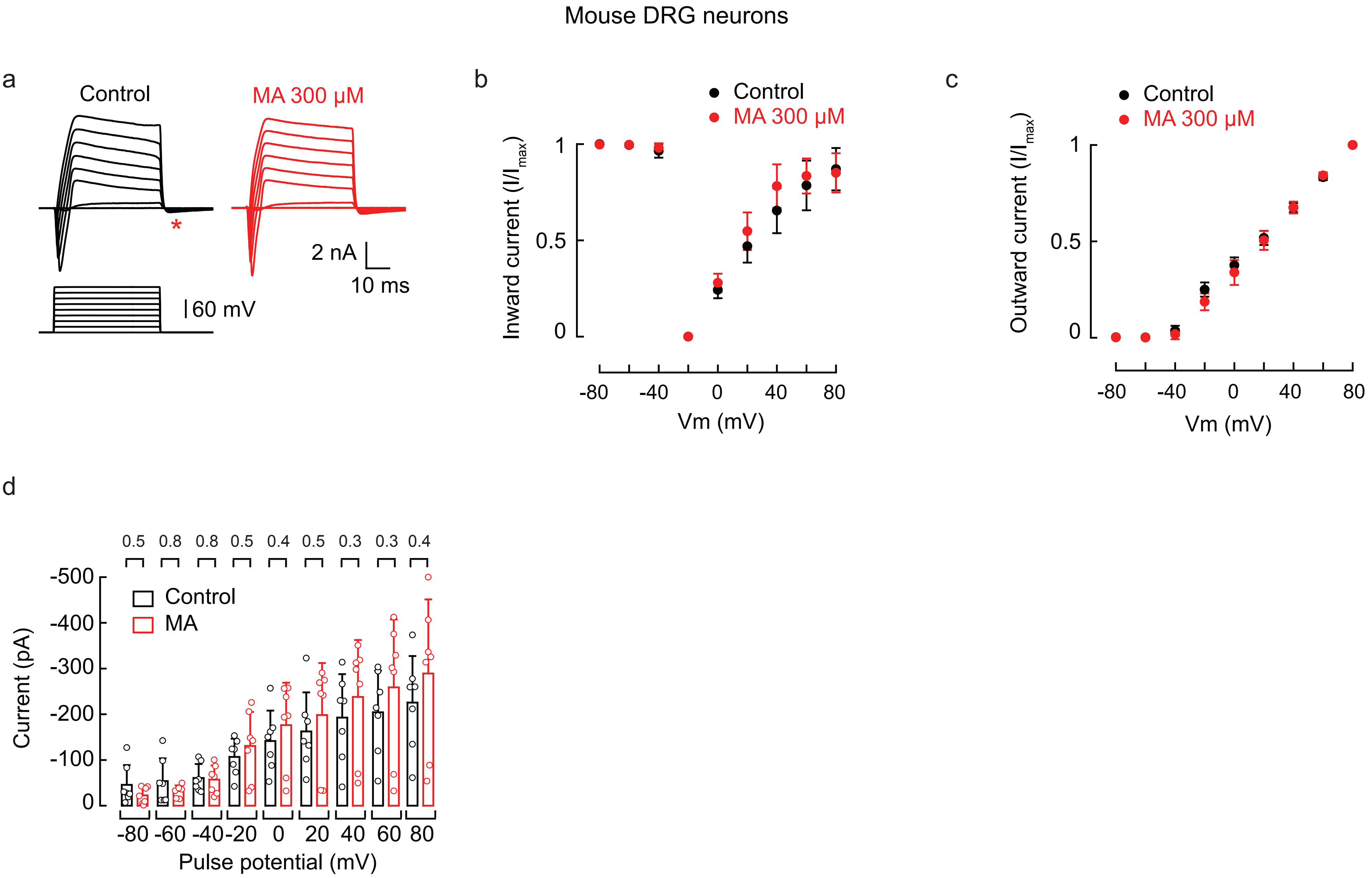

Mouse DRG neurons
a
Control
MA 300 μM
*
2 nA
10 ms
60 mV
b
Control
MA 300 μM
Inward current (I/I max)
1
0.5
0
-80
-40
0
40
80
Vm (mV)
c
Control
MA 300 μM
Outward current (I/I max)
1
0.5
0
-80
-40
0
40
80
Vm (mV)
d
0.5
0.8
0.8
0.5
0.4
0.5
0.3
0.3
0.4
Control
MA
Current (pA)
-500
-400
-300
-200
-100
0
-80
-60
-40
-20
0
20
40
60
80
Pulse potential (mV)

Supplementary figure 5. Related to figure 4.

a Representative whole-cell patch-clamp recordings of control and MA (300 μM)-treated mouse DRG neurons depolarized in a stepwise manner from a holding potential of -80 mV. Red asterisk denotes the time interval selected to determine the current magnitude after the voltage-pulse protocol.

b Normalized inward current densities elicited by stepwise depolarization from a holding potential of -80 mV of control and MA (300 μM)-treated mouse DRG neurons. (n = 7). Circles are mean ± SD.

c Normalized outward current densities elicited by stepwise depolarization from a holding potential of -80 mV of control and MA (300 μM)-treated mouse DRG neurons. (n = 7). Circles are mean ± SD.

d Peak currents within the first 10 ms interval after the end of the voltage protocol (Red asterisk) *vs.* pulse potential from control and MA (300 μM)-treated mouse DRG neurons (n = 7). Bars are mean ± SD. Two-tailed Mann-Whitney test for -80, -60, 20, 40, and 60, and two-tailed unpaired t-test for -40, -20, 0, and 80.

p values are denoted above the bars.

Supplementary Figure 6

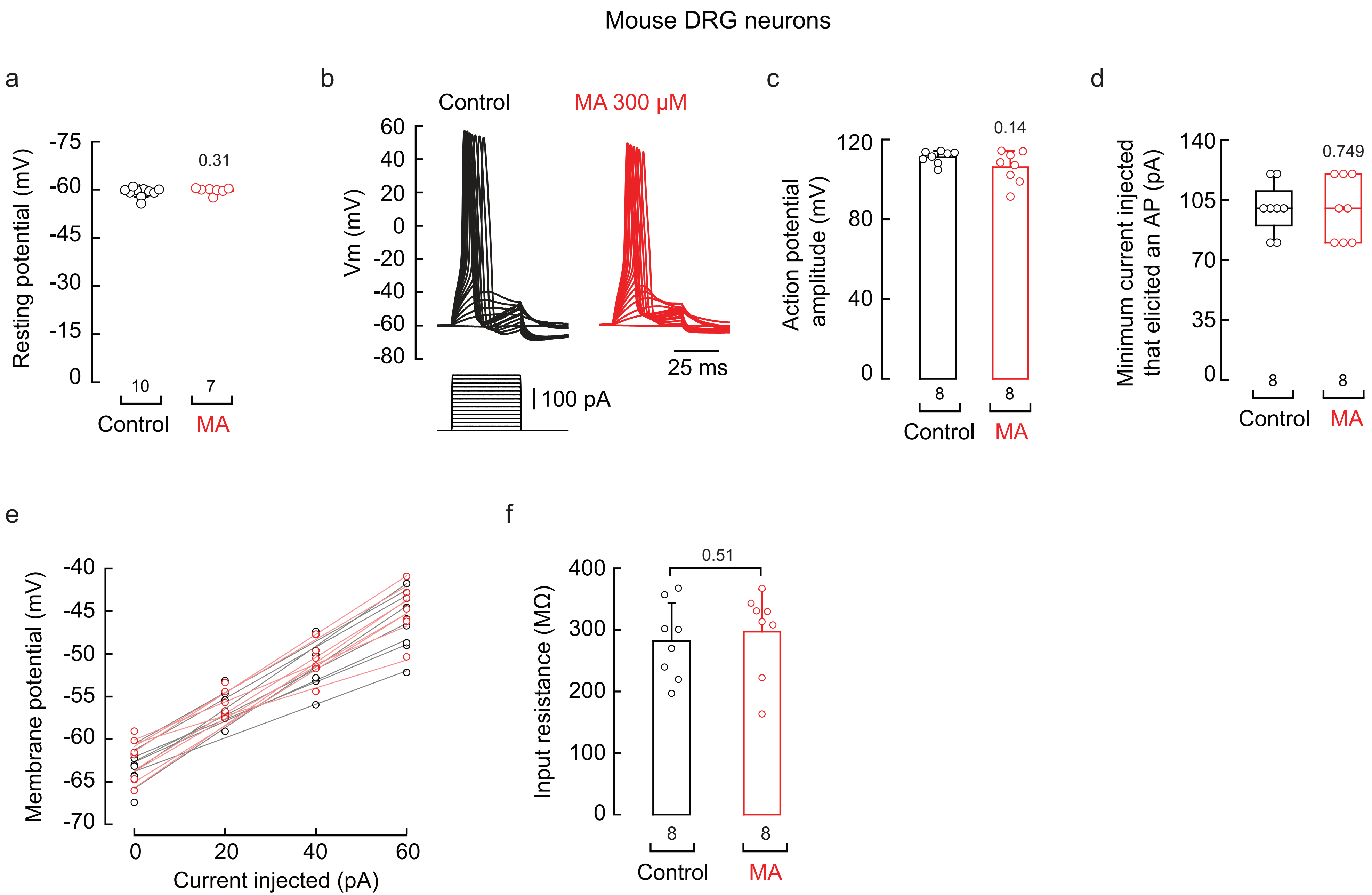

Supplementary figure 6. Related to figure 4.

a Membrane potential values recorded just after the whole-cell configuration was achieved from control and MA (300 µM)-treated mouse DRG neurons. n is denoted above the *x*-axis. Two-tailed Mann-Whitney test.

b Representative current-clamp recording of membrane potential changes elicited by current injection in control and MA (300 µM)-treated mouse DRG neurons.

c Action potential amplitudes evoked by current injection from control and MA (300 µM)-treated mouse DRG neurons. Bars are mean ± SD. n is denoted above the *x*-axis. Two-tailed unpaired t-test with Welch's correction.

d Minimum current injected to elicit action potentials in control and MA (300 µM)-treated mouse DRG neurons. Two-tailed permutation t-test.

e Membrane potential changes elicited by stepwise current injections of control (black) and MA (red; 300 µM)-treated mouse DRG neurons. n = 8.

f Input resistance of control and MA (300 µM)-treated mouse DRG neurons. Input resistance was calculated as the slope of linear fits of current-voltage plots (shown on a) generated from a series of increasing current injection steps1. n is denoted above the *x*-axis. Two-tailed Mann-Whitney test.

p values are denoted above the bars and box.

Mouse DRG neurons

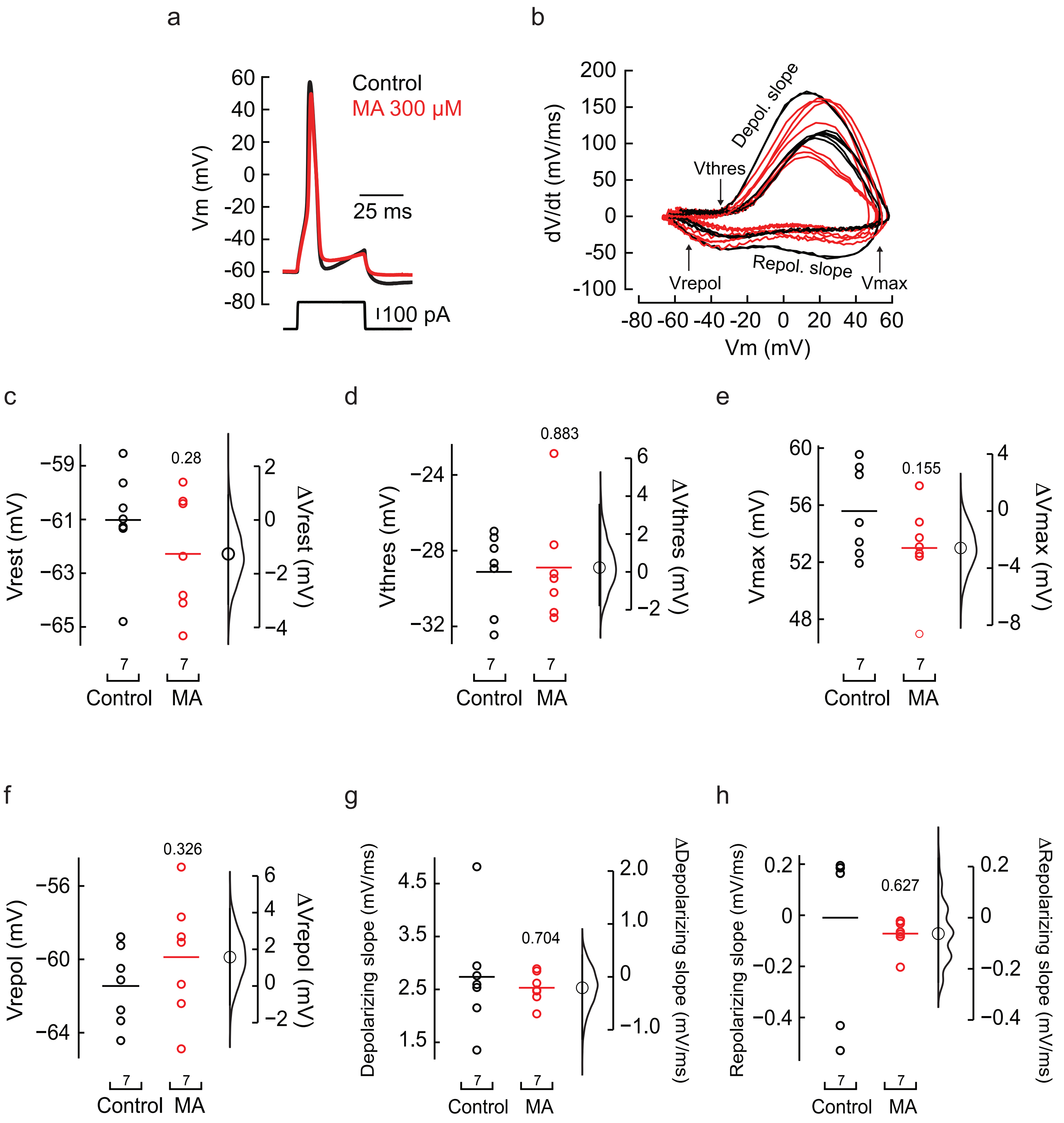

Supplementary figure 7. Related to figure 4.

a Current-clamp recordings of membrane potential changes elicited by current injection (280 pA) in control and MA (300 µM)-treated mouse DRG neurons.

b Phase plot (*d*V/*d*t *vs.* V) analysis of action potentials depicted in *a*.

c-h Mean differences between control and MA (300 µM)-treated mouse DRG neurons are shown in the above Gardner-Altman estimation plots[1] for the resting and threshold membrane potentials (Vrest and Vthres, respectively), maximal voltage peak (Vmax), the repolarization potential (Vrepol), and the depolarization and repolarization slopes from each AP trace shown in *a*. Experimental groups are plotted on the left axes; the mean differences are plotted on floating axes on the right as a bootstrap sampling distribution. The mean differences are depicted as a dot; the 95% confidence interval is indicated by the ends of the vertical error bar. n is denoted above the *x*-axis. *p* values were determined with two-tailed permutation *t*-test and are denoted above the plots.

Supplementary Figure 8

Rat DRG neurons

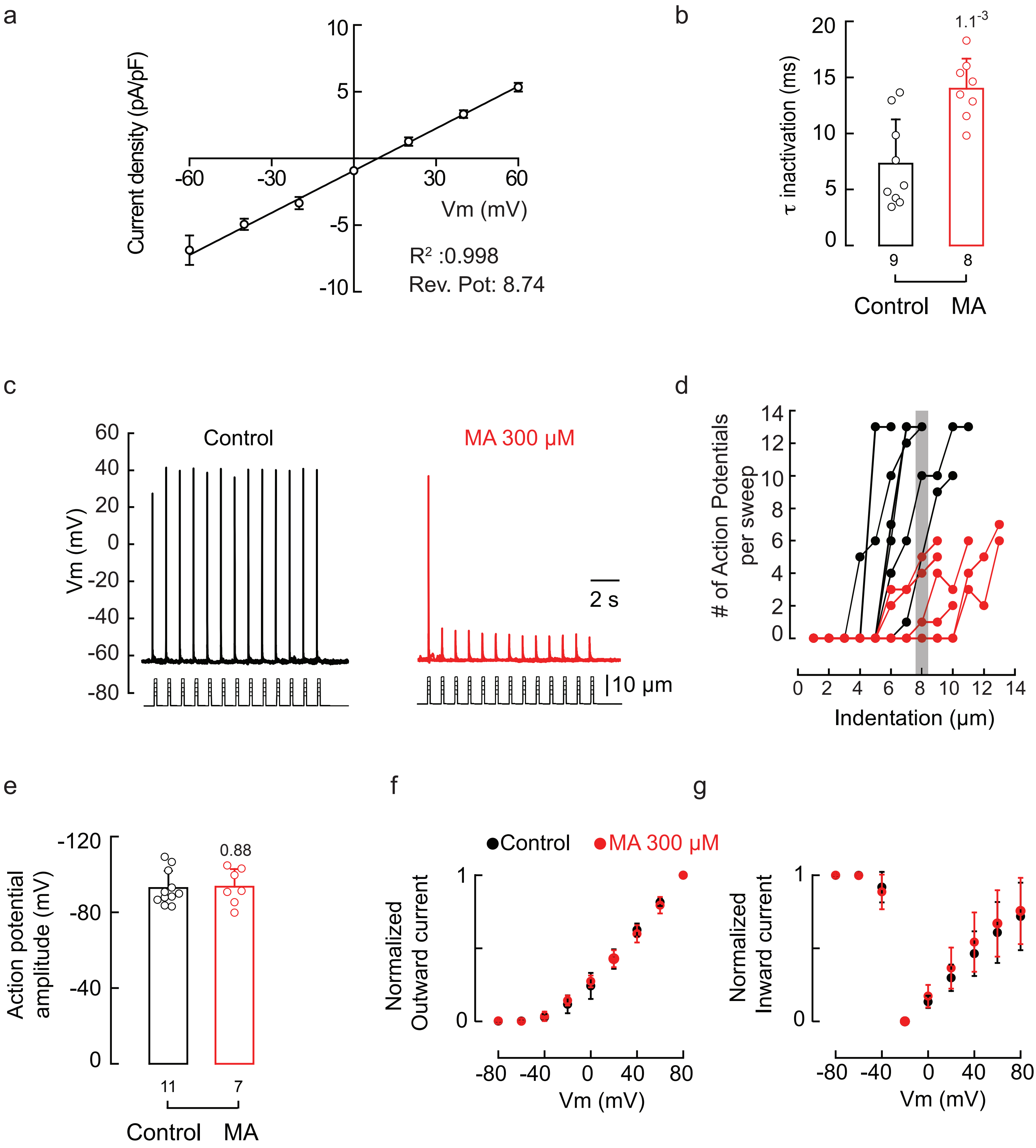

Supplementary figure 8. Related to figure 5.

a Current-voltage relationship of rat DRG neuron mechano currents as determined by whole-cell patch-clamp experiments. Circles are mean ± SD. n = 3.

b Time constant of inactivation of currents elicited by maximum displacement of control and MA (300 µM for 18h)-treated rat DRG neurons. Bars are mean ± SD. n is denoted above the *x*-axis. Two-tailed unpaired t-test

c Representative current-clamp recordings of membrane potential changes elicited by a train of mechanical pulses of control (black) and MA-treated (red) rat DRG neurons.

d Number of action potentials elicited per sweep *vs.* mechanical indentation of control (black; n = 6) and MA-treated (red; n = 6) treated rat DRG neurons.

e Action potential amplitude measured from resting potential to peak membrane potential of control and MA (300 µM for 18h,)-treated rat DRG neurons. Bars are mean ± SD. n is denoted above the *x*-axis. Two-tailed unpaired t-test.

f Normalized outward current densities elicited by stepwise depolarization from a holding potential of -80 mV of control (n = 8) and MA (n = 6; 300 µM)-treated rat DRG neurons. Circles are mean ± SD.

g Normalized inward current densities elicited by stepwise depolarization from a holding potential of -80 mV of control (n = 8) and MA (n = 6; 300 µM)-treated rat DRG neurons. Circles are mean ± SD.

p values are denoted above the bars.

Supplementary Figure 9

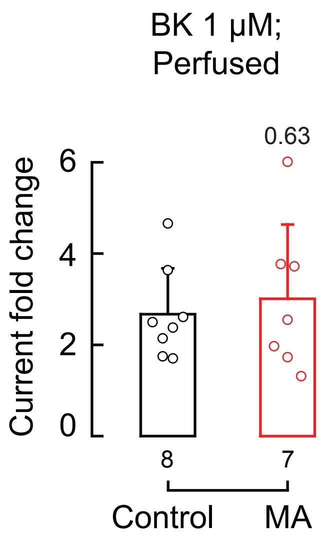

Supplementary figure 9. Related to figure 6.

Current fold change of control and MA (300 μM; 18h)-treated mouse DRG neurons perfused for 60 s with bath solution and 300 s with bath solution containing Bradykinin (BK; 1 μM) consecutively. Bars are mean ± SD. n is denoted bellow bars. Two-tailed unpaired t-test.

p value is denoted above the bar.

Supplementary Figure 10

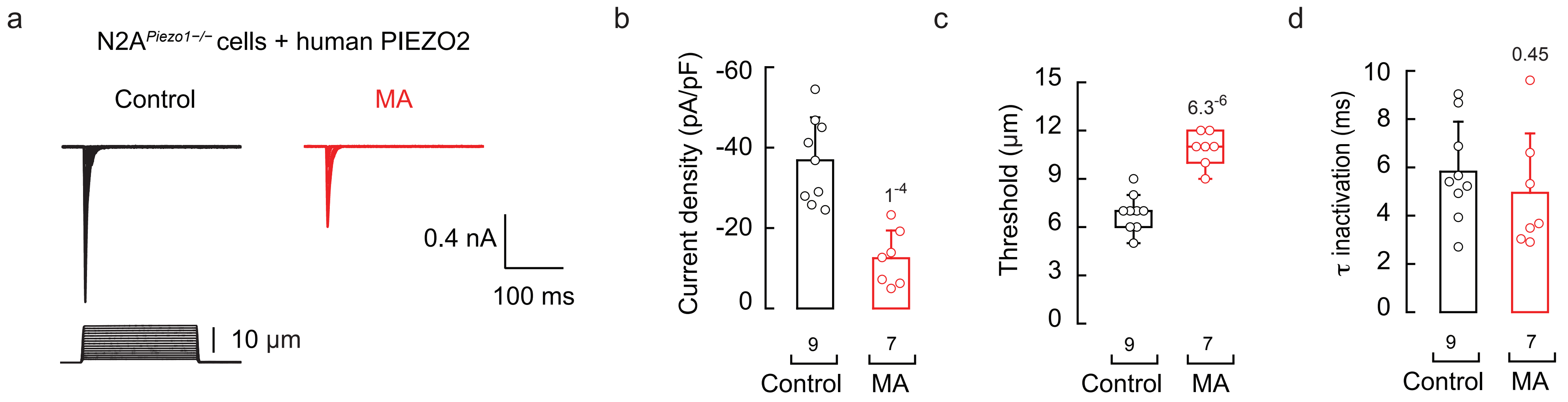

Supplementary figure 10. Related to figure 7.

a Representative whole-cell patch-clamp recordings elicited by mechanical stimulation (at -60 mV) of control and MA (300 μM)-supplemented N2A$^{Piezo1-/-}$ cells transfected with human *Piezo2*.

b Current densities elicited by maximum displacement of control and MA (300 μM; 18 h)-treated N2A$^{Piezo1-/-}$ cells transfected with human *Piezo2*. Bars are mean ± SD. n is denoted above the *x*-axis. Two-tailed unpaired t-test.

c Displacement thresholds required to elicit currents of control and MA (300 μM; 18 h)-treated N2A$^{Piezo1-/-}$ cells transfected with human *Piezo2*. Boxplots show mean (square), median (bisecting line), bounds of box (75th to 25th percentiles), outlier range with 1.5 coefficient (whiskers), and minimum and maximum data points. n is denoted above the *x*-axis. Two-tailed unpaired t-test.

d Time constants of inactivation elicited by maximum displacement of control and MA (300 μM; 18h)-treated N2A$^{Piezo1-/-}$ cells transfected with human *Piezo2*. Bars are mean ± SD. n is denoted above the *x*-axis. Two-tailed unpaired t-test.

p values are denoted above the bars.

Table 1. Mean values ± SD of V_{rest}, V_{thres}, V_{max}, and V_{repol}, and depolarizing and repolarizing action potential slopes.

AP properties	Control	MA (300 µM)	*p* value
Resting membrane potential ($V_{rest,}$ mV)	-61.02 ± 0.69	-62.28 ± 1.88	0.280
Threshold membrane potential (V_{thres}, mV)	-29.12 ± 2.16	-28.89 ± 3.56	0.883
Maximal voltage peak (V_{max}, mV)	55.58 ± 2.67	52.99 ± 3.86	0.155
Repolarization potential (V_{repol}, mV)	-61.45 ± 1.83	-59.88 ± 3.75	0.326
Depolarizing slope (mV/ms)	2.73 ± 1.06	2.53 ± 0.31	0.704
Repolarizing slope (mV/ms)	-0.01 ± 0.32	-0.07 ± 0.06	0.627

Action potential properties were determined from phase plots on Supplementary Figure 7. *p* values were determined with two-sided permutation *t*-test[1]. n = 7 for control and MA-treated DRG neurons.

References

1 Ho, J., Tumkaya, T., Aryal, S., Choi, H. & Claridge-Chang, A. Moving beyond P values: data analysis with estimation graphics. *Nat Methods* **16**, 565-566, doi:10.1038/s41592-019-0470-3 (2019).
