# Supplementary material for: A dietary fatty acid counteracts neuronal mechanical sensitization
Source: Nat Commun. 2020 Jun 19;11:2997. doi: 10.1038/s41467-020-16816-2 (PMC7305179; doi:10.1038/s41467-020-16816-2)
Supplement: Supplementary file 3 — Reporting Summary [file 41467_2020_16816_MOESM3_ESM.pdf]

nature research

Corresponding author(s): Valeria Vásquez, Ph.D.

Last updated by author(s): May 15, 2020

Reporting Summary

Nature Research wishes to improve the reproducibility of the work that we publish. This form provides structure for consistency and transparency in reporting. For further information on Nature Research policies, see Authors & Referees and the Editorial Policy Checklist .

Statistics

For all statistical analyses, confirm that the following items are present in the figure legend, table legend, main text, or Methods section.

n/a	Confirmed	
☐	☒	The exact sample size (*n*) for each experimental group/condition, given as a discrete number and unit of measurement
☐	☒	A statement on whether measurements were taken from distinct samples or whether the same sample was measured repeatedly
☐	☒	The statistical test(s) used AND whether they are one- or two-sided<br>*Only common tests should be described solely by name; describe more complex techniques in the Methods section.*
☐	☒	A description of all covariates tested
☐	☒	A description of any assumptions or corrections, such as tests of normality and adjustment for multiple comparisons
☐	☒	A full description of the statistical parameters including central tendency (e.g. means) or other basic estimates (e.g. regression coefficient) AND variation (e.g. standard deviation) or associated estimates of uncertainty (e.g. confidence intervals)
☐	☒	For null hypothesis testing, the test statistic (e.g. *F*, *t*, *r*) with confidence intervals, effect sizes, degrees of freedom and *P* value noted<br>*Give P values as exact values whenever suitable.*
☒	☐	For Bayesian analysis, information on the choice of priors and Markov chain Monte Carlo settings
☒	☐	For hierarchical and complex designs, identification of the appropriate level for tests and full reporting of outcomes
☒	☐	Estimates of effect sizes (e.g. Cohen's *d*, Pearson's *r*), indicating how they were calculated

Our web collection on statistics for biologists contains articles on many of the points above.

Software and code

Policy information about availability of computer code

Data collection	For electrophysiology: Clampex v10.4.2.0 (Molecular Devices, LLC).
Data analysis	For electrophysiology: Clampfit v10.4.2.0 (Molecular Devices, LLC), for plotting OriginPro 2018 (64-bit) v:b9.5.1.195 (OriginLab Coorporation), and for statistical analyses: GraphPad Instat v.3.10.32 (GraphPad Software, Inc) and EstimationStats (Open software and code available in: estimationstats.com; property of Adam Claridge-Chang and Joses Ho).

For manuscripts utilizing custom algorithms or software that are central to the research but not yet described in published literature, software must be made available to editors/reviewers. We strongly encourage code deposition in a community repository (e.g. GitHub). See the Nature Research guidelines for submitting code & software for further information.

Data

Policy information about availability of data

All manuscripts must include a data availability statement. This statement should provide the following information, where applicable:

- Accession codes, unique identifiers, or web links for publicly available datasets
- A list of figures that have associated raw data
- A description of any restrictions on data availability

Data supporting the findings of this manuscript are available from the corresponding author upon reasonable request. A reporting summary for this article is available as a Supplementary Information file. The source data underlying Figures and Supplementary Figures are provided as a Source Data file, DOI: 10.6084/m9.figshare.12192630

Field-specific reporting

Please select the one below that is the best fit for your research. If you are not sure, read the appropriate sections before making your selection.

[x] Life sciences [ ] Behavioural & social sciences [ ] Ecological, evolutionary & environmental sciences

For a reference copy of the document with all sections, see nature.com/documents/nr-reporting-summary-flat.pdf

Life sciences study design

All studies must disclose on these points even when the disclosure is negative.

Sample size	No sample-size calculation was performed. For electrophysiology: we obtained data for more than 4 samples per condition (at least three different days). The sample sizes were deemed sufficient based on studies that used similar methodologies.
Data exclusions	For electrophysiological experiments we excluded from the analyses (exclusion criteria were pre-established): (1) cells with leak currents bigger than 200 pA, (2) cells that detached from the substrate during mechanical stimulation and (3) cells which giga-seals did not withstand at least 6 indentation steps of the protocol. These criteria were implemented in order to avoid introducing artifacts with the mechanical stimulation protocols implemented, and including recordings where the voltage-clamp was unstable.
Replication	All attempts at replication were successful. Electrophysiology experiments were performed at least 3 times in different days with different preparations.
Randomization	Daily measurements included the control and treated samples. For electrophysiological experiments only cells with good surface attachment were chosen (for example: healthy morphology and visible pseudopodia). Transfected cells had a fluorescent transfection marker; and cells that endogenously express PIEZO2 (neurons and MCC13 cells) were chosen randomly. Cell size was taken into account by normalizing each cell's current magnitude (pA) by their capacitance (pF; which is proportional to the cell size).
Blinding	The investigators were not blind during data acquisition and analysis. Electrophysiological recordings involved monitoring of time lapses during treatments that made blinding not feasible.

Reporting for specific materials, systems and methods

We require information from authors about some types of materials, experimental systems and methods used in many studies. Here, indicate whether each material, system or method listed is relevant to your study. If you are not sure if a list item applies to your research, read the appropriate section before selecting a response.

Materials & experimental systems

n/a	Involved in the study	
[x]	[ ]	Antibodies
[ ]	[x]	Eukaryotic cell lines
[x]	[ ]	Palaeontology
[ ]	[x]	Animals and other organisms
[x]	[ ]	Human research participants
[x]	[ ]	Clinical data

Methods

n/a	Involved in the study	
[x]	[ ]	ChIP-seq
[x]	[ ]	Flow cytometry
[x]	[ ]	MRI-based neuroimaging

Eukaryotic cell lines

Policy information about cell lines

Cell line source(s)	Neuro-2a (N2A, ATCC CCL-131) from ATCC; Piezo1 knock-out mouse N2A cells were a gift from Dr. Gary Lewin (produced from N2A, ATCC CCL-131 from ATCC; Reference: Moroni, M., Servin-Vences, M.R., Fleischer, R. et al. Voltage gating of mechanosensitive PIEZO channels. Nat Commun 9, 1096 (2018). https://doi.org/10.1038/s41467-018-03502-7); MCC13 cells were purchased from Sigma Aldrich; rat dorsal root ganglia neurons were purchased from Cell-Applications; and human iPSC-derived neurons were generated from a WTC11 iPSC line (Coriell Institute GM25256).
Authentication	None of the cell lines used were authenticated in the lab.
Mycoplasma contamination	All cell lines tested negative for mycoplasma contamination.
Commonly misidentified lines (See ICLAC register)	No commonly misidentified cell lines were used in the study.

Animals and other organisms

Policy information about studies involving animals; ARRIVE guidelines recommended for reporting animal research

Laboratory animals	8-12-week-old male C57BL/6 mice (Mus musculus).
Wild animals	No wild animals were used in the study.
Field-collected samples	No field collected samples were used in the study.
Ethics oversight	Protocols for mice have been reviewed and approved by the IACUC. The University of Tennessee maintains an AALAC-accredited facility that is staffed by 3 full time veterinarians, 2 of whom are board-certified in laboratory animal care. Protocols for human iPSC-derived neurons were reviewed and approved by approved by the IRB at the National Institute of Neurological Disorders and Stroke Intramural Research Program.

Note that full information on the approval of the study protocol must also be provided in the manuscript.
